# Methods in the design and implementation of the Randomized Evaluation of Sedation Titration for Respiratory Failure (*RESTORE*) clinical trial

**DOI:** 10.1186/s13063-018-3075-8

**Published:** 2018-12-17

**Authors:** Martha A. Q. Curley, Rainer G. Gedeit, Brenda L Dodson, June K. Amling, Deborah J. Soetenga, Christiane O. Corriveau, Lisa A. Asario, David Wypij, For the RESTORE Investigative Team

**Affiliations:** 1 School of Nursing, University of Pennsylvania, Philadelphia, PA USA; 2 Perelman School of Medicine at the University of Pennsylvania, Philadelphia, PA USA; 3 Research Institute, Children’s Hospital of Philadelphia, Philadelphia, PA USA; 4 Department of Pediatrics, Medical College of Wisconsin, Milwaukee, WI USA; 5 Critical Care Section, Children’s Hospital of Wisconsin, 9000 W. Wisconsin Avenue, MS #681, Milwaukee, WI 53226 USA; 6 Information Services Department, Boston Children’s Hospital, Boston, MA USA; 7 Department of Plastic Surgery, Children’s National Health System, Washington, DC USA; 8 Pediatric Intensive Care Unit, American Family Children’s Hospital, Madison, WI USA; 9 Department of Critical Care Medicine, Children’s National Medical Center, Washington, DC USA; 10 Department of Cardiology, Boston Children’s Hospital, Boston, MA USA; 11 Department of Biostatistics, Harvard School of Public Health, Boston, MA USA; 12 Department of Pediatrics, Harvard Medical School, Boston, MA USA

**Keywords:** Cluster randomized design, Treatment fidelity, Pilot study, Nurse-led therapy, Goal-directed therapy, Trajectory of illness, Algorithms, Competing trials

## Abstract

**Background:**

Few papers discuss the pragmatics of conducting large, cluster randomized clinical trials. Here we describe the sequential steps taken to develop methods to implement the Randomized Evaluation of Sedation Titration for Respiratory Failure (*RESTORE)* trial that tested the effect of a nurse-implemented, goal-directed, comfort algorithm on clinical outcomes in pediatric patients with acute respiratory failure.

**Methods:**

After development in a single institution, the *RESTORE* intervention was pilot-tested in two pediatric intensive care units (PICUs) to evaluate safety and feasibility. After the pilot, the *RESTORE* intervention was simplified to enhance reproducibility across multiple PICUs. The final *RESTORE* trial was developed as a cluster randomized clinical trial where the unit of randomization was the PICU, stratified by PICU size, and the unit of inference was the patient. Study execution was revised based on our Data and Safety Monitoring Board’s recommendation to consult with the Department of Health and Human Services’ Office of Human Research Protection (OHRP) on how best to consent eligible subjects. OHRP deemed that the *RESTORE* intervention posed greater than minimal risk and that all enrolled subjects provide consent reflecting their level of participation.

**Results:**

Thirty-one PICUs of varying size, organization and academic affiliation participated and over 2800 critically ill infants and children supported on mechanical ventilation for acute pulmonary disease were enrolled. The primary outcome for the trial was the duration of mechanical ventilation; secondary outcomes included time awake and comfortable, total sedative exposure and iatrogenic withdrawal symptoms. Throughout the clinical trial the investigative team worked to maintain treatment fidelity, enrollment milestones and co-investigator enthusiasm. We considered the potential impact of competing clinical trials through a decision-making framework.

**Conclusions:**

The *RESTORE* clinical trial was a large and complex multicenter study that has provided the necessary evidence to guide sedation practices in the field of pediatric critical care. Specific issues that were unique to this trial included level of consent, adding clinical sites to augment enrollment and evaluating the potential impact of competing clinical trials.

**Trial registration:**

ClinicalTrials.gov, Identifiers: Pilot trial: NCT00142766; Retrospectively registerd on 2 September 2005.

Cluster randomized trial: NCT00814099. Registered on 23 December 2008.

## Background

Providing sedation to help comfort critically ill infants and children supported on mechanical ventilation is a routine aspect of pediatric intensive care [1–3]. However, sedative exposure is associated with iatrogenic injury [4–12]; specifically, sedatives may depress spontaneous ventilation and prolong the duration of mechanical ventilation, may impair the neurological examination necessitating diagnostic testing and, when discontinued, may precipitate iatrogenic withdrawal syndrome (IWS) prolonging hospital stay [13–19]. In adult intensive care, data have supported a shift in sedation goals from an unresponsive patient to a calm, easily aroused, readily evaluated, critically ill patient [20–22]. Sedation strategies tested in adult intensive care units (ICUs) include daily prospective identification of a sedation endpoint, nurse-implemented sedation protocols that include daily arousal assessments and/or titration of sedation, synchronizing level of sedation with the ventilator strategy and spontaneous breathing trials. After hospital discharge, post-traumatic stress disorder occurs less frequently in more awake adult ICU patients managed with daily sedative interruptions [23, 24].

Without data, clinician bias impacts local sedation practices with a net effect of wide variation in sedation management practices across all pediatric intensive care units (PICUs) [1]. To address this lack of pediatric-specific data, we performed the Randomized Evaluation of Sedation Titration for Respiratory Failure (*RESTORE*) clinical trial [25]. *RESTORE* was a multicenter cluster randomized clinical trial that tested the effect of a pediatric sedation management protocol on clinical outcomes in pediatric patients with acute respiratory failure. Here we describe the sequential steps taken to develop the methods and implement *RESTORE* that tested the effect of a nurse-implemented, goal-directed, comfort algorithm on clinical outcomes in pediatric patients with acute respiratory failure. We discuss the importance of pilot-testing and scaling up a clinical intervention, consideration of the level of consent in a cluster randomized trial, methods to augment enrollment and assessing potential impact of competing clinical trials.

## Methods

### Development of the *RESTORE-beta* protocol

A multidisciplinary task force from the medical-surgical pediatric intensive care unit at Boston Children’s Hospital led by MAQC designed and implemented eight successive drafts of the pediatric-specific sedation protocol (*RESTORE-beta*) over a 2-year period (1999–2001) [26]. Unique features of the protocol included matching the therapeutic sedation goal to the individual patient’s trajectory of illness, mandated sedation titration every 8 h in the non-acute phase, a rapid opioid wean followed by a slow benzodiazepine wean and the use of methadone only in patients with IWS complicating recovery [27].

### Pilot-testing *RESTORE-beta*

From 2003 to 2006, we performed a two-center, stepped-wedge pilot test of *RESTORE-beta* in children supported on mechanical ventilation for acute respiratory failure in two PICUs: Children’s National Medical Center, Washington, DC and Children’s Hospital Wisconsin, Milwaukee, WI (R21 HD045020; Curley). For this trial each institution implemented *RESTORE-beta* as standard of care for sedation management in all children with primary respiratory failure. Both PICUs provided baseline data on their sedation practices for 3 months. After that, one PICU was randomized to start the intervention phase while the second PICU continued usual care. After 9 months, the second PICU implemented *RESTORE-beta* while protocol sustainability was evaluated in the first PICU. Both units used the same sedation, pain and withdrawal scoring assessment tools and extubation readiness test. Each PICU implemented *RESTORE-beta* as a unit-based standard of care and data were collected in a subset of patients with primary pulmonary disease. Inclusion and exclusion criteria are shown in Table 1. The study was reviewed and approved by the coordinating center and local Institutional Review Boards. Legal guardians provided informed consent for data collection in one PICU and the local Institutional Review Board waived the need for informed consent in the other PICU.

**Table 1:** Randomized Evaluation of Sedation Titration for Respiratory Failure (*RESTORE*) eligibility criteria

Inclusion criteria:	
• ≥ 2 weeks of age, ≥ 42 weeks post-menstrual age, and < 18 years of age	
• Supported on mechanical ventilation for acute lung disease. Lung disease includes both airways and parenchymal disease	
Exclusion criteria:	
• Cyanotic heart disease with unrepaired or palliated right to left intracardiac shunt	
• History of single ventricle at any stage of repair	
• Congenital diaphragmatic hernia or congenital/acquired diaphragm paralysis	
• Primary pulmonary hypertension	
• Critical airway (e.g., post laryngotracheal reconstruction) or anatomical obstruction of the lower airway (e.g., mediastinal mass)	
• Ventilator dependent (including non-invasive) on PICU admission (chronic assisted ventilation)	
• Neuromuscular respiratory failure	
• Spinal cord injury above the lumbar region	
• Pain managed by patient-controlled analgesia (PCA) or epidural catheter	
• Patient transferred from an outside ICU where sedatives had already been administered for more than 24 h	
• Family/medical team have decided not to provide full support (patient treatment considered futile)	
• Enrolled in any other critical care interventional clinical trial concurrently or within the last 30 days	
• Known allergy to any of the study medications	
• Pregnancy	

Over 27 months, 2095 pediatric patients supported on mechanical ventilation were screened and data were collected from 245 patients who met study criteria. Enrolled patients were 2.4 years of age (median; IQR 0.5–11.3 years), 54% male, with predominately normal cognitive and functional health [28]. Common reasons for mechanical ventilation included pneumonia (38%), bronchiolitis (17%) and thoracic trauma (10%). Their Pediatric Risk of Mortality III (PRISM III) score was 9 (median; IQR 5–17), with an associated risk of mortality of 5% (median; IQR 2–22%) [29].

The primary efficacy outcome for this pilot trial was duration of mechanical ventilation. Median duration of mechanical ventilation decreased during the intervention phase at both sites (141 to 107 h at site 1 and 177 to 162 h at site 2). Adjusting for age group, PRISM III score, Pediatric Outcome Performance Category (POPC) at enrollment and site, the estimated common adjusted hazard ratio was 1.24 between the intervention and baseline phases (95% confidence interval 0.91–1.67; *P* = 0.17), which corresponded to a 19% (− 10 to 40%) reduction in median duration of mechanical ventilation.

From a safety perspective, the combined unplanned extubation rate for the two sites during the intervention phase was 2.6/100 ventilator days, which was within the range of published rates (0.25 to 3.0/100 ventilator days) [30–34]. Arousal assessments were performed in 13 patients and lasted 290 min (median; IQR 75–2160 min). One patient did not awaken during an assessment, which prompted a neurological evaluation that identified an intracranial hemorrhage. There were also trends toward decreased PICU and hospital lengths of stay, less opioid exposure and fewer days of opioid exposure during the intervention phase.

Based on these pilot data and clinician debriefing after the pilot, we made the following changes to *RESTORE-beta*:

- Improve care provider buy-in and facilitate implementation by assuring medical and nursing PICU leadership support using a signed contract showing support
- Improve protocol compliance by emphasizing the *RESTORE* goal to ensure an awake yet comfortable patient using the lowest effective dose of a limited number of analgesics and sedatives for the shortest period of time
- Enhance treatment fidelity by linking the arousal assessment to the patient’s sedation level
- Rename the “plateau phase” the “titration phase” to reinforce that the goal of that phase of the protocol is to titrate, not maintain comfort medication infusions
- Avoid confusion in drug titration by simplifying the algorithm by matching the frequency of drug titration (every 8 h) in the titration and weaning to extubation phases
- Assure compliance with extubation assessment by modifying the extubation readiness test when the patient is spontaneously breathing with an oxygenation index of ≤ 6. (Use a SpO_2_ – Estimated PaO_2_ conversion table to estimate the PaO_2_ in patients without an arterial line.)
- Make dose titration consistent by changing the rate of opioid weaning from 20% every 12 h to 10% every 8 h in the weaning phase

### *RESTORE* – Phase III clinical trial

During study design, we rejected the idea of performing a before/after trial as we did in the pilot because changes in usual PICU care could occur over time and impact study outcomes. We also considered randomization by patient, team and PICU. We rejected patient-level randomization because the intervention required a PICU-wide practice change in how clinical teams work together. Patient randomization could lead to group contamination over time and be disruptive to unit operations since bedside teams could be responsible for caring for patients in each group at the same time. We rejected team randomization because multiple teams rotate in a PICU over the course of a day. We chose cluster randomization by PICU to limit contamination between groups. In addition, a multicenter clinical trial allows potential bias to be distributed across diverse practice settings, allows the comprehensive assessment of patient risk, allows the determination of the level of protocol compliance necessary to assure desired patient outcomes and increases the generalizability of study results. We therefore developed the multicenter, cluster randomized clinical trial testing the *RESTORE* protocol on the duration of mechanical ventilation in pediatric patients supported on mechanical ventilation for acute respiratory failure (U01 HL086622 and HL086649; Curley and Wypij).

We selected a parallel cluster randomized clinical trial design rather than a stepped-wedge design because (1) there were no data to support the *RESTORE* intervention and (2) PICUs were hesitant to implement an untested intervention. The hybrid stepped-wedge cluster randomized trial (intervention delivered in half the PICUs randomized to the intervention in a stepped-wedge fashion with the remainder functioning as controls) could have been selected as an alternative to the parallel cluster study though we predicted that the PICUs were relatively homogeneous with a small intra-cluster correlation coefficient (ICC) so that our parallel study would tend to deliver better statistical performance than a stepped-wedge trial. Logistically, we were also prepared to start all intervention sites at approximately the same time.

Initially we designed the study using the PICU as the unit of randomization where all patients in the randomized PICU would receive the intervention and provide deidentified information. However, during this time, the Department of Health and Human Services’ Office of Human Research Protection (OHRP) produced opinions around risks to subjects enrolled in clinical trials [35]. Based on the newly published recommendations, the *RESTORE* Data and Safety Monitoring Board (DSMB) asked that we contact OHRP to opinion on our planned strategy and need for informed consent. OHRP then opinioned that *RESTORE* was a research intervention that posed more than minimal risk and that all parents/legal guardians should provide informed consent. Therefore, *RESTORE* was implemented as a parallel cluster randomized controlled trial where PICUs assigned to the intervention group implemented the *RESTORE* protocol as a research intervention only on consented subjects while PICUs randomized to the control group continued their usual sedation practices and data were collected only on consented subjects.

The *RESTORE* intervention was an organizational change directed at all PICU clinicians and included:

1. Team education on the use of the sedation protocol in pediatric patients supported on mechanical ventilation
2. Team identification of the patient’s trajectory of illness and daily prescription of a sedation goal
3. A nurse-implemented, goal-directed, comfort algorithm that guides moment-to-moment titration of opioids and benzodiazepines
4. Team feedback on sedation management performance

The unit of randomization was the PICU, the unit of inference was the patient and we controlled for center effects.

The study was reviewed and approved by the DSMB and all local Institutional Review Boards, including those of the clinical and data coordinating centers. Legal guardians of all study participants provided written informed consent using one of three separate consent documents (baseline phase, usual care, or intervention) that described the cluster randomized design and the risk associated with their level of participation (data collection with/without intervention). Inclusion and exclusion criteria were unchanged from the pilot study (Table 1). Screening was performed at least once daily in each PICU to identify potential subjects. Daily data collection occurred in all enrolled subjects from endotracheal intubation until the end of their scheduled sedation therapy, hospital discharge, or day 28 (whichever occurred first). Half of all enrolled subjects continued to be followed at approximately 6 months post PICU discharge for comparison of long-term outcomes. The primary outcome of this Phase III clinical trial was the duration of mechanical ventilation. Secondary outcomes are shown in Table 2.

**Table 2:** Randomized Evaluation of Sedation Titration for Respiratory Failure (*RESTORE*) secondary outcome variables

• Time to recovery of acute respiratory failure (from endotracheal intubation to first meeting criteria to be tested for extubation readiness)	
• Duration of weaning from mechanical ventilation (from first meeting criteria to be tested for extubation readiness to first successful extubation – defined as extubation for more than 24 h)	
• Occurrence of adverse events: inadequate pain management, inadequate sedation management, clinically significant iatrogenic withdrawal symptoms, unplanned extubation, airway irritation from movement of the endotracheal tube within the airway, extubation failure/reintubation within 24 h of extubation, dislodgement of vascular access or drainage tubes, ventilator-associated pneumonia (VAP)^a^, catheter-associated blood stream infection (CA-BSI)^a^, and stage 2+ pressure ulcers. Report of a new critical airway will be assessed through hospital discharge or day 90 (whichever occurs first)	
• Detection of life-threatening neurological events	
• Occurrence of iatrogenic withdrawal symptoms	
• PICU and hospital LOS	
• Hospital costs	
• Protocol implementation costs	
• Cost-effectiveness	
• In-hospital mortality	
• Post-discharge quality of life and emotional health	

^a^The National Nosocomial Infections Surveillance System (NNIS) definitions will be used to define VAP and CA-BSI. All cases of VAP and CA-BSI will be adjudicated by a process outlined by Cook et al. [47]. *LOS* length of stay, *PICU* pediatric intensive care unit

### Collaborating centers

Clinical sites were recruited from the Pediatric Acute Lung Injury and Sepsis Investigators (PALISI) Network. Participating PICUs mock screened their unit for 1 month to evaluate their available patient population and completed an organizational assessment describing their participating unit structure, work processes, change processes and sedation practices. Organizations with more than one PICU selected one participating PICU that housed their general medical patients. All participating PICUs (1) verified that they did not have a sedation protocol in place, (2) showed evidence of critical care nursing and physician leadership support, (3) agreed to implement the same pain, sedation and withdrawal assessment instruments and (4) could enroll a minimum of three subjects per month. Because of the cluster randomized design, the participants needed to agree to the research design where PICUs would be randomized to the *RESTORE* intervention as a research protocol while the remaining PICUs continued usual care and would continue to maintain their current practices in the control PICUs. During the pre-randomization period, all PICUs provided baseline data on their usual sedation practices and provided approximately 3 months of baseline data so that site comparability at trial entry could be evaluated. A natural grouping of PICUs emerged based on the number of eligible patients: small, medium and large.

In 2009, the study began with 22 sites randomized (12 intervention and 10 control). Because of the need to obtain consent in control PICUs where only data collection was occurring versus the intervention units where a change in practice was being performed, we anticipated lower consent rates in the intervention PICUs. Based on our previous experience, we anticipated a consent rate of > 85% in control PICUs and a consent rate of 60% in intervention PICUs [36, 37]. Because of these estimates, we randomized more sites to the intervention arm. Study enrollment was subsequently reviewed in June 2010. Enrollment rates from these 22 sites were slightly lower than expected. To increase enrollment rates, we added nine sites in 2010, following the same site selection procedures and randomization scheme as for the original sites. Specifically, we recruited them from the PALISI Network, performed an organizational assessment, classified them by size and randomized them by size, five to the intervention arm and four to the control arm.

### Study procedures

Study procedures were focused to enhance treatment fidelity as follows. Each participating center identified a physician-nurse-pharmacist co-investigator team responsible for discipline-specific education, compliance assessment and retraining. After randomization, co-investigators from the intervention sites attended a start-up meeting and completed a competency-based training program and certification process prior to enrolling intervention patients. This process included review of the web-based Manual of Operations and study videos.

After the start-up meeting, intervention PICUs conducted team training for 1 month and then implemented the *RESTORE* sedation protocol on consented patients. Team training included all clinicians (physicians, nurses, clinical pharmacists, respiratory therapists) involved in the management of intubated and mechanically ventilated patients. Training materials included discipline-specific slide packages, informal case discussions, a video of the nurse-implemented, goal-directed, comfort algorithm and arousal assessment, pocket reminder cards and bedside booklets. Prior to the intervention phase, all physicians, unit-based clinical pharmacists and nursing staff were required to demonstrate their understanding of the intervention by completion of a discipline-specific, scenario-based self-assessment evaluation requiring a score of ≥ 80%. If a score was < 80%, retraining was required, and the assessment repeated until the score was ≥ 80%. In addition to the core physician-nurse-pharmacist team, additional multidisciplinary “Champions” served as unit-based resources for the *RESTORE* protocol. Champions provided clinicians access to a *RESTORE* protocol expert at all times.

During routine daily multidisciplinary patient care rounds, the protocol directs clinicians to identify the patient’s trajectory of illness and prescribe a daily sedation goal. The bedside nurse then used the algorithm with complementary standardized order set to titrate the comfort medications to the prescribed sedation target. The order set served three purposes: (1) reinforcement of training, (2) decreased delay in implementing a change in sedation and (3) enhanced protocol compliance.

The decision to require consent for each intervention patient impacted the trial by providing staff utilizing *RESTORE* with less experience using the protocol. Because of this we required a minimal enrollment of three subjects per month and a rigorous training and quality improvement plan. To assure protocol compliance, reinforce education and assess safety during the intervention period, one site co-investigator rounded separately on each study patient each day. During these investigator rounds, the co-investigator or nurse champion offered staff support and retraining as necessary and completed a “Walk Rounds Report” that collected data around these topics and issues. Any deficits identified during walk rounds was addressed with the care team to assure compliance with the protocol. The reports were summarized weekly to provide team feedback on *RESTORE* performance.

### Post-discharge quality of life and emotional health

Follow-up was conducted on a stratified random sample of approximately half of the consented subjects. To ensure that the sample was representative of all subjects in the trial, the sample was stratified by study site and age. Consenting families were sent a reminder letter and copies of the follow-up assessment instruments by mail and called 6 months (± 1 month) after PICU discharge.

We monitored our follow-up rates monthly and increased our sampling ratio when necessary to meet expected milestones. Follow-up rates did not differ by treatment arm. Treatment group differences in patients with follow-up were similar to those in the main trial, with patients in the intervention arm being younger and having lower risk of mortality, less frequent history of asthma and a different distribution of primary diagnosis category [38].

### Statistical considerations

The PICUs were block randomized by size (small, medium and large) to assure a balanced allocation between the control and intervention groups.

With respect to the primary outcome, duration of mechanical ventilation, subjects were assigned 28 days if they were still intubated after 28 days or if they were transferred or died prior to day 28 without remaining extubated for > 24 h prior to transfer/death. This mortality-adjusted duration of mechanical ventilation is a continuous variable that is effectively equivalent to ventilator-free days [39]. We anticipated that the mortality rate in the first 28 days would be low and similar between the control and intervention groups. If that was not the case in the study, we would have performed a secondary analysis excluding these deaths from the analysis, effectively comparing the duration of mechanical ventilation among survivors. If the mortality rate was higher than anticipated and/or is unbalanced between control and intervention groups, we planned to conduct a competing risks analysis, treating extubation and death as two competing events.

The primary analysis compared the duration of mechanical ventilation in intervention and control subjects using Kaplan-Meier survival curves and proportional hazards regression adjusting for age group, PRISM III score and POPC at enrollment. We considered PICU as a cluster variable in the survival analyses using Lin and Wei’s sandwich variance estimator [40].

### Study monitoring

An independent DSMB monitored the clinical trial for adverse events, adherence to study protocol and potential early stopping. To determine if early stopping was warranted, group sequential monitoring was used to assess for efficacy and the method of stochastic curtailment was used to assess for futility. The sample size was adjusted using an O’Brien-Fleming stopping rule to accommodate three formal interim analyses after approximately 400, 1200 and 1800 subjects [41].

### Sample size and power considerations

We hypothesized that patients managed with a pediatric sedation management protocol would experience a shorter duration of mechanical ventilation compared with those not managed with the sedation protocol. The study team determined that a 20% reduction in the duration of mechanical ventilation was clinically important. This clinically important 20% reduction, or hazard ratio of 1.25, was plausible based on the results of the pilot study.

Assuming independent observations and proportional hazards between treatment groups, 892 events (successful extubations) were required for a two-sided, 0.05-level log-rank test to achieve 90% power to detect a 20% reduction in ventilation duration with the intervention assuming three interim analyses (East, Version 5.3, Cytel Statistical Software, Cambridge, MA, USA). As we expected no more than 15% of the subjects to be censored at 28 days given our experience with the prone positioning study [37] and the pilot sedation management study, we required 1050 subjects to achieve this power. However, because observations from subjects at the same site may be correlated, we inflated this sample size to account for the ICC in our cluster randomized trial design [42]. The ICC for the Martingale residuals of extubation times was estimated to be 0.00 in the seven-site prone-positioning study [37] and 0.01 in the two-site pilot sedation management study. Conservatively based on the first 22 *RESTORE* sites, using ICC = 0.01 leads to 1990 total subjects needed. Increasing the number of sites or number of subjects will increase the power. We chose 2448 enrolled subjects as our target sample size, guaranteeing 90% power to detect a 20% reduction in length of extubation controlling for censoring, three formal interim analyses for early stopping and modest within-site correlations.

### Quality control

Pediatric critical care nurses with research experience uniquely served as study monitors and conducted site visits, beginning at least 6 months after the start of the intervention phase of the trial. The design of the trial required that intervention sites implement the intervention on consented subjects and that control sites not implement the protocol. We monitored the intervention in the intervention sites and monitored aspects of the protocol in the control sites to assess protocol drift. All visits included an observation during multidisciplinary rounds, spot check of interrater reliability on assessment tools and an audit of at least 10% of randomly selected data. To maintain competency in study procedures, sites were required to enroll a minimal number of patients per month. If enrollment dropped under the minimum for three consecutive months, the site was required to implement a quality improvement plan that included physician and nurse retraining.

### Competing trials

During the *RESTORE* clinical trial, there were several other large, multicenter clinical trials recruiting similar patients in the PALISI Network [43, 44]. As investigators, we sought every opportunity to share potential subjects while maintaining study integrity. The impact of co-enrollment on *RESTORE* and each competing trial was systematically evaluated by creating a “Co-Enrollment Decision-Making Grid” that summarized the shared population, enrollment window, timing of the intervention and duration of study, (Table 3). We also considered the study endpoints; specifically, any overlapping primary and/or secondary outcomes. If possible, we described the magnitude of the interaction of the two interventions; for example, whether one intervention would dilute or enhance the effect of the other intervention. We considered the level of randomization (patient or site) and the potential for enrollment imbalance at each site and in total; for example, enrolling only septic patients with acute respiratory failure thus removing them from *RESTORE*. We considered the potential impact on data interpretation and whether we had sufficient power to evaluate potential interactions. From a safety perspective, we evaluated whether the specified adverse events could be clearly attributed to either or both interventions. Finally, we considered the potential consent burden placed on parents/legal guardians, and the workload burden placed on site investigators and/or the bedside team. The completed Co-Enrollment Decision-Making Grid along with a recommendation from the Steering Committee was then sent to the DSMB and NIH for their review and final recommendation [25, 45–48]. With *RESTORE*, observational studies were typically approved, and interventional studies were not. If co-enrollment was not allowed, a local randomization scheme avoided clinician bias on what study to offer consecutive eligible parents/legal guardians.

**Table 3:** Co-enrollment decision-making grid

	Impact of co-enrollment	Trial A	Trial B
Scientific integrity			
Intervention-related			
1. Population of concern	Overlap?		
2. Enrollment window	Which closes first?		
3. Timing of intervention	Overlap?		
4. Study period	Overlap?		
5. Exclusion criteria	Conflict?		
Overlapping endpoints			
6. Primary endpoint	Overlap?		
	Potential impact?		
7. Secondary and exploratory endpoints	Overlap?		
	Potential impact?		
Other			
8. Magnitude of interaction	Known/unknown?		
	Dilution/enhancement of effect?		
9. Level of randomization	Same/different?		
10. Timing of randomization	Same/different?		
11. Potential of imbalance	Yes/no?		
12. Effect of co-enrollment	Contamination?		
Data interpretation			
13. Power to determine interactions	Sufficient/insufficient?		
14. Attribution of adverse events	Easy/difficult?		
Feasibility/burden			
15. Parent/legal guardian burden	Yes/no?		
16. Site investigator burden	Yes/no?		
17. Bedside clinical team burden	Yes/no?		
18. Current sharing scheme	Yes/no?		
Additional considerations			
19. Impact on publication	Known/unknown?		
20. Intervention use off-protocol	Yes/no?		
21. Priority	High/low?		
22. Possible sharing arrangement			

## Results

The *RESTORE* clinical trial was successfully completed in 2013 and the results of the primary trial and secondary manuscripts have been published with others in development using the data set obtained in this large clinical trial [25, 49–54]. Briefly, the trial demonstrated that the *RESTORE* intervention did not impact the duration of mechanical ventilation but could be safely implemented by nurses at multiple sites and reduce sedative exposure while keeping patients in a more awake state than those in the control group.

## Discussion

Here we describe the development, pilot-testing, final design and implementation of the *RESTORE* clinical trial so that other clinical trialist can better understand its genesis, preliminary testing, rationale and challenges in performing an appropriately powered, complex clinical trial in pediatric critical care. The study began in June 2009 and ended in December 2013. We enrolled 309 patients during the baseline phase, 59 patients while intervention sites were completing training, and 2449 patients during the intervention phase with 1225 in the intervention arm and 1224 in the control arm. The primary and numerous secondary papers have been published [25, 45–49].

Pilot-testing *RESTORE-beta* was an important step in the research process to assure that a protocol, developed at one site, could be successfully implemented at unique sites. The pilot study allowed for the evaluation of the safety and efficacy of the protocol, education methods and study governance needed for the subsequent large, complex clinical trial. This step was key in developing the strategies to assure compliance, such as daily walk rounds and immediate education and/or clarification for the bedside care team and site visits from clinical monitors.

The study was initially designed as a cluster randomized clinical trial using the PICU as the unit of randomization with all patients in the randomized PICU receiving the intervention. At its first meeting, the DSMB requested that OHRP be consulted to advise on the appropriate level of consent. The DSMB determined that OHRP opinions around risks to subjects enrolled in clinical trials produced at the time of this protocol development could affect protocol design. Once opinioned that the intervention posed more than minimal risk, the investigative team redesigned the trial so that the intervention was only applied to consented subjects in PICUs randomized to receive the intervention. This opinion avoided local controversy and variation on the level of consent. The decision impacted the trial by providing staff in the randomized PICU less experience with the protocol. We accommodated this by requiring a minimal enrollment of three subjects per month and implementing a rigorous training and quality improvement plan if this benchmark was not met.

The evaluation of enrollment numbers during the trial allowed for recruitment of additional sites so that the study could be completed on time. Little information is available about how to add sites to a cluster randomized clinical trial. Important considerations included the process of identifying sites, avoiding selection bias and maintaining consistent training processes.

The decision to evaluate the effect of competing trials was important for *RESTORE* and other clinical trials performed during the same time period. The decisions to prioritize enrollment and discuss co-enrollment strategies allowed successful completion of *RESTORE* and other pivotal trials [43, 44].

Rather than seeking an elusive ideal drug, the *RESTORE* protocol focuses on optimal clinical decision-making. This study tests an explicit team approach for sedation management, with an emphasis on maintaining a minimum yet effective dose of sedation while minimizing iatrogenic injury. Since the *RESTORE* protocol provides a team approach to help improve sedation management of critically ill infants and children it can potentially be used with multiple combinations of drugs used to manage comfort in intubated children.

## Conclusions

The *RESTORE* trial was successfully completed and the primary and numerous secondary papers have been published. The data have the potential to significantly improve the care of critically ill infants and children by developing a generalizable strategic approach that optimizes patient comfort and tolerance of invasive support. We describe the development, pilot-testing, design and implementation of the *RESTORE* clinical trial so that clinicians can better understand its genesis, preliminary testing, rationale and challenges in performing an appropriately powered complex clinical trial using cluster randomization. Specific issues that investigators should consider when developing clinical trials include the level of consent, plans for adding clinical sites to augment enrollment and dealing with competing clinical trials.

## Abbreviations

DSMB: Data and Safety Monitoring Board
ICC: Intra-cluster correlation
ICUs: Adult intensive care units
IWS: Iatrogenic withdrawal syndrome
OHRP: Office of Human Research Protection
PALISI: Pediatric Acute Lung Injury and Sepsis Investigators
PICU: Pediatric intensive care unit
POPC: Pediatric Overall Performance Category
PRISM III: Pediatric Risk of Mortality III
*RESTORE*: Randomized Evaluation of Sedation Titration for Respiratory Failure
RESTORE-beta: Protocol used in pilot study
