# Correction to: Methods in the design and implementation of the Randomized Evaluation of Sedation Titration for Respiratory Failure (RESTORE) clinical trial

**DOI:** 10.1186/s13063-018-3154-x

**Published:** 2019-01-07

**Authors:** Martha A. Q. Curley, Rainer G. Gedeit, Brenda L. Dodson, June K. Amling, Deborah J. Soetenga, Christiane O. Corriveau, Lisa A. Asaro, David Wypij, For the RESTORE Investigative Team

**Affiliations:** 1 School of Nursing, University of Pennsylvania, Philadelphia, PA USA; 2 Perelman School of Medicine at the University of Pennsylvania, Philadelphia, PA USA; 3 Research Institute, Children’s Hospital of Philadelphia, Philadelphia, PA USA; 4 Department of Pediatrics, Medical College of Wisconsin, Milwaukee, WI USA; 5 Critical Care Section, Children’s Hospital of Wisconsin, 9000 W. Wisconsin Avenue, MS #681, Milwaukee, WI 53226 USA; 6 Information Services Department, Boston Children’s Hospital, Boston, MA USA; 7 Department of Plastic Surgery, Children’s National Health System, Washington, DC USA; 8 Pediatric Intensive Care Unit, American Family Children’s Hospital, Madison, WI USA; 9 Department of Critical Care Medicine, Children’s National Medical Center, Washington, DC USA; 10 Department of Cardiology, Boston Children’s Hospital, Boston, MA USA; 11 Department of Biostatistics, Harvard School of Public Health, Boston, MA USA; 12 Department of Pediatrics, Harvard Medical School, Boston, MA USA

**Correction to: Trials (2018) 19:687**

**https://doi.org/10.1186/s13063-018-3075-8**

Following publication of the original article [1], the authors notified us of a typing error in spelling Dr. Asario’s name. The original publication has been corrected.

- Originally published name:
  Lisa A. Asario
- Corrected name:
  Lisa A. Asaro
